# Supplementary material for: Deletion of wfs1 Impairs Oligodendrocyte Precursor Cells Dorsal Distribution and Myelination Through the wfs1-hmgcs1 Axis in Zebrafish
Source: Biology (Basel). 2026 Aug 16;15(16):1402. doi: 10.3390/biology15161402 (PMC13510151; doi:10.3390/biology15161402)
Supplement: Supplementary file 1 [file biology-15-01402-s001.zip › File S1-Transcriptome sequencing data and differentially expressed genes (DEGs) in wfs1-- zebrafish at 5 dpf/2.KEGG/wfs1KOvsWT/ALL/src/dre04810.html]

dre04810


- dre:445305

- Down regulated genes

445305\_1(-1.98867996431405)

- dre:767646

- Down regulated genes

767646\_1(-3.30449808454776)

- dre:100002872

- Up regulated genes

100002872\_2(4.61563278220247)

- dre:566764

- Up regulated genes

566764\_1(9.05474774224364)

- Down regulated genes

566764(-2.60134676797662)
- dre:323285

- Down regulated genes

323285\_1(-3.43280624877224)

- dre:286739

- Up regulated genes

286739(1.28316470710399)

- Down regulated genes

286739\_1(-2.60068066374091)

- dre:30429

- Up regulated genes

30429(3.98975094921512)

- Down regulated genes

30429\_2(-1.78714384880949)
- dre:677750

- Up regulated genes

677750(1.37913343503877)

- dre:321070

- Up regulated genes

101882911(3.50047007681359)

- dre:553493

- Up regulated genes

553493\_1(1.65608040669544)

- Down regulated genes

553493(-1.0476065195569)

- dre:793158

- Up regulated genes

793158\_1(1.86160461223548)

- Down regulated genes

793158(-6.94943122774273)
- dre:556226

- Up regulated genes

556226\_1(6.36622858283894)

- dre:792699

- Down regulated genes

792699(-1.84389282152821)

- dre:436718

- Up regulated genes

436718(1.34229427553561)

- Down regulated genes

436718\_1(-6.85571323128554)

- dre:564565

- Up regulated genes

564565(1.26204775967321)

- dre:100000252

- Up regulated genes

100000252\_3(1.94434154854557) 100000252(1.09163460881879)

- Down regulated genes

100000252\_1(-1.7718817168434)

- dre:58075

- Up regulated genes

58075(1.04367086328776)

- dre:100331581

- Up regulated genes

100331581(2.2819785165305)

- Down regulated genes

100331581\_1(-6.65159438165074)

- dre:570868

- Up regulated genes

570868\_1(5.31304404972279)
- dre:557935

- Down regulated genes

557935(-1.87319484154862)
- dre:100536705

- Down regulated genes

100536705\_2(-6.17198352976631)
- dre:797491

- Down regulated genes

797491\_1(-1.09418588609297)
- dre:564266

- Up regulated genes

564266(2.42311476837666)

- dre:100005469

- Up regulated genes

100005469\_1(3.66353583031119)

- dre:386856

- Down regulated genes

386856\_1(-1.99487147218035) novel.946(-6.7676180476835)
- dre:569566

- Down regulated genes

569566(-1.00953785382545)
- dre:571431

- Down regulated genes

571431(-6.42862549702529)

- dre:100005049

- Down regulated genes

100005049(-1.30748784788409)
- dre:560564

- Down regulated genes

560564\_2(-4.02056550705436)
- dre:796490

- Up regulated genes

796490\_2(1.58582846768856)
- dre:494456

- Up regulated genes

494456(1.65469795112832)
- dre:103909601

- Up regulated genes

103909601(4.16535952448207)

- Down regulated genes

378480(-1.33145438810737)

- dre:794365

- Up regulated genes

794365(1.73411710545423)

- dre:57934

- Up regulated genes

57934\_1(9.91304974129429)

- Down regulated genes

57934(-1.25539807424297)

- dre:57934

- Up regulated genes

57934\_1(9.91304974129429)

- Down regulated genes

57934(-1.25539807424297)

- dre:57934

- Up regulated genes

57934\_1(9.91304974129429)

- Down regulated genes

57934(-1.25539807424297)

- dre:445305

- Down regulated genes

445305\_1(-1.98867996431405)

- dre:799354

- Up regulated genes

799354(4.08509859637481)

- Down regulated genes

799354\_1(-1.21394100025012)

- dre:570832

- Up regulated genes

570832(3.62075873481994) 570832\_1(3.61949176292455)
- dre:393611

- Down regulated genes

393611(-2.47215503276962)
- dre:798694

- Up regulated genes

798694\_1(3.42103651644023)

Close
